# Genomic Characterization of *Leishmania tropica* in Cutaneous Leishmaniasis, Somali Region, Ethiopia, 2023

**DOI:** 10.3201/eid3107.241607

**Published:** 2025-07

**Authors:** Adugna Abera, Pieter Monsieurs, Myrthe Pareyn, Dereje Beyene, Geremew Tasew, Allison Aroni-Soto, Mahlet Belachew, Desalegn Geleta, Bethlehem Adnew, Bokretsion Gidey, Henok Tadesse, Atsbeha Gebreegziabxier, Rajiha Abubeker, Abraham Ali, Ketema Tafess, Tobias F. Rinke de Wit, Johan van Griensven, Jean-Claude Dujardin, Dawit Wolday, Malgorzata Anna Domagalska

**Affiliations:** College of Natural and Computational Sciences, Addis Ababa University, Addis Ababa, Ethiopia (A. Abera, D. Beyene); Ethiopian Public Health Institute, Addis Ababa (A. Abera, G. Tasew, M. Belachew, D. Geleta, B. Gidey, H. Tadesse, A. Gebreegziabxier, R. Abubeker, A. Ali, D. Wolday); Institute of Tropical Medicine, Antwerp, Belgium (P. Monsieurs, M. Pareyn, A. Aroni-Soto, J. van Griensven, J.-C. Dujardin, M.A. Domagalska); Armauer Hansen Research Institute, Addis Ababa (B. Adnew); Adama Science and Technology University, Adama, Ethiopia (K. Tafess); Amsterdam Institute for Global Health and Development, Amsterdam University Medical Centre, University of Amsterdam, Amsterdam, the Netherlands (T.F.R. de Wit); Michael G. DeGroote Institute for Infectious Disease Research, Hamilton, Ontario, Canada (D. Wolday); McMaster Immunology Research Center, Health Sciences, McMaster University, Hamilton (D. Wolday)

**Keywords:** cutaneous leishmaniasis, vector-borne infections, parasites, Leishmania tropica, zoonoses, genomic surveillance, outbreak, Somali, Ethiopia

## Abstract

We sequenced *Leishmania tropica* genomes from 8 human skin samples collected in a newly emerging focus of cutaneous leishmaniasis in the Somali region of Ethiopia. We found a variant with unique genomic signatures of drug resistance. Public health officials should use genomic surveillance to slow expansion of *L. tropica*.

Since the successful Kala-Azar elimination program in the Indian subcontinent, the hotspot of worldwide leishmaniasis has moved to East Africa (*1*). Among the different affected countries, Ethiopia deserves particular attention, given the heterogeneous eco-epidemiology of leishmaniasis, its clinical polymorphism, and the complex taxonomy of *Leishmania tropica* parasites. The disease is endemic in different biotopes from lowlands to highlands, and transmission involves different hosts and vectors (*2*). The 4 major clinical forms of leishmaniasis are visceral leishmaniasis (VL), causing 2,500–4,000 reported cases, and 3 forms of cutaneous leishmaniasis (CL), localized, diffuse, and muco-cutaneous, causing ≈50,000 reported cases. *L. donovani* (VL and occasionally CL) and *L. aethiopica* (all 3 CL forms) are the most reported species, and *L. tropica* (CL) was isolated once from a human patient (*2*); several interspecies hybrids have been observed (*3*).

The epidemiology of the disease is affected by human migration and displacement because of famine and regular conflicts in the country and by environmental changes. We previously highlighted the need for genomic surveillance of leishmaniasis by using highly sensitive, resolutive, and untargeted whole-genome sequencing (WGS) methods (*4*) for the following reasons: since the discovery of hybridization and genetic introgression (*5*), robust species identification should theoretically be on the basis of multigenic approaches covering several regions of the genome (and not only single gene approaches); WGS is needed to assess the genetic similarity among parasites sampled from different patients, thereby confirming the outbreak nature of a focus; and WGS can be used to find signatures of drug resistance and guide patient management.

In August 2023, an outbreak of CL was detected among immunologically naive militia recently deployed in the eastern Somali region of Ethiopia. That area did not have a previous history of CL, but sporadic cases of VL were reported. The clinical manifestations of the CL cases (multiple wet lesions) was not comparable to what is typically observed in Ethiopia and neighboring countries (single dry lesions). Hsp70 amplicon sequencing identified the CL pathogen as *L. tropica* (A. Abera et al., unpub. data, https://www.medrxiv.org/content/10.1101/2024.10.05.24314933v1).

We undertook a more in-depth molecular characterization of *L. tropica* samples collected in the focus in the Somali region (Figure 1). We used direct genome sequencing of *Leishmania* in host tissues (SureSelect sequencing; Agilent Technologies, https://www.agilent.com) that did not require parasite isolation and cultivation (*6*). That method was previously validated for *L. donovani* in bone marrow (*6*) and blood samples (*7*), and we used it for the first time for skin samples from CL patients.

**Figure 1:**
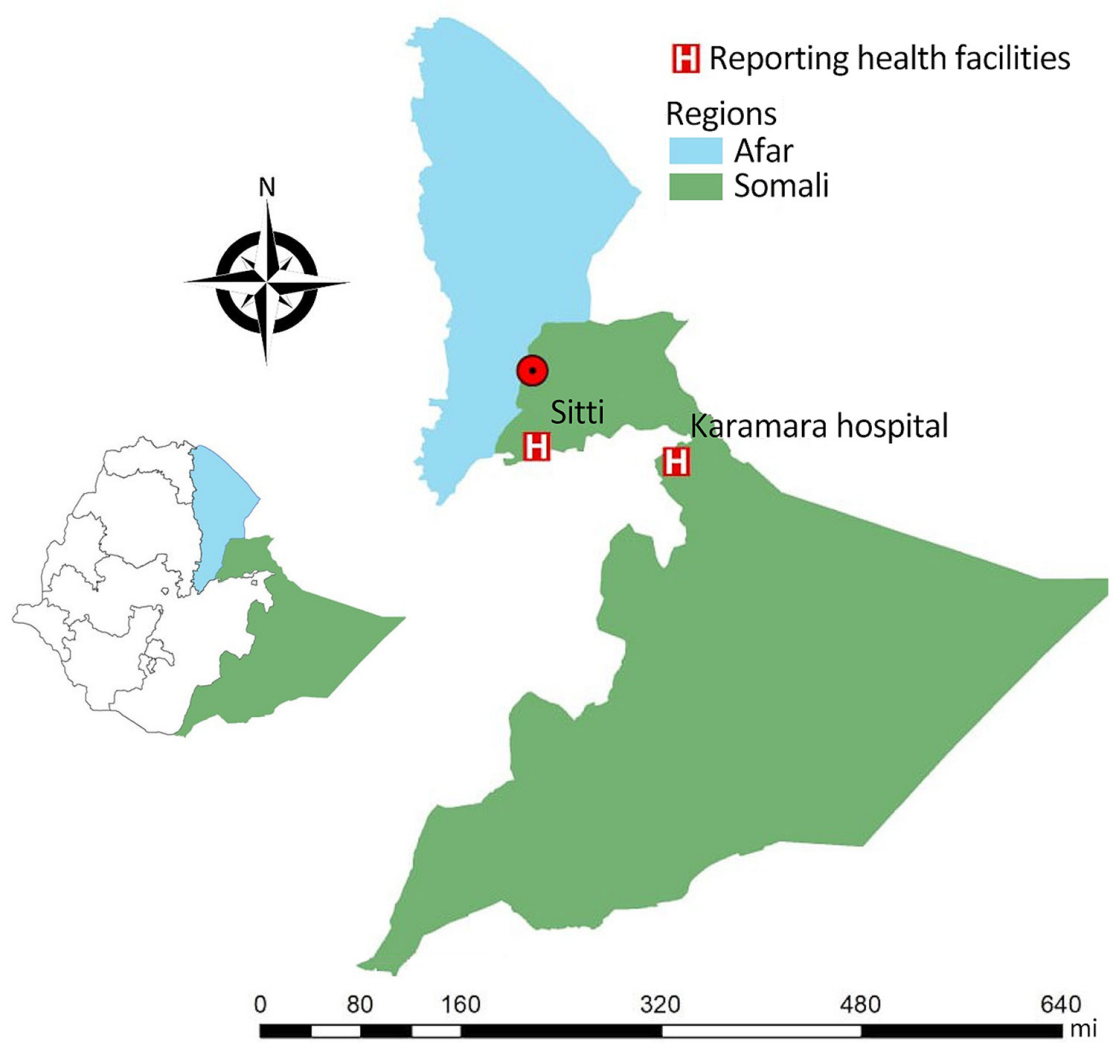
Locations of the healthcare facilities (HF) for the 8 study patients in genomic characterization of *Leishmania tropica* in cutaneous leishmaniasis, Somali region, Ethiopia, 2023. Map of Ethiopia shows the Afar region in blue and the Somali region in green. Two cases (S04 and S06) were diagnosed at Duunyar Health Center, located at the border of the Afar and Somali regions, where the outbreak occurred (red dot). One case (S12) was diagnosed at Sitti Primary Hospital, and 5 cases (WH08, WH09a, WH11, WH12, and WH15) were reported at Jigjiga University Sheik Hassen Yabare Comprehensive Specialized Hospital (CSH), each marked by a red H.

We submitted 8 of the CL samples from the Somali region for SureSelect sequencing by using a capture panel of probes designed for the *L. aethiopica* genome. SureSelect sequencing should work well with phylogenetically related species such as *L. tropica*. We used competitive mapping (Appendix Figure 1) and phylogenetic analysis (Appendix Figure 2) for the species identification of the 8 samples. Those samples (Appendix Figure 2, yellow arrow) clearly branch in the *L. tropica* cluster and are genetically very different from *L. aethiopica*, *L. major*, *L. donovani*, and interspecies hybrids. 

In a second step, we only focused on *L. tropica* genomes (Figure 2). That focus provided 4 major insights. The 8 Somali region parasites constitute a *L. tropica* variant not previously reported in analyzed genomes. Those variants form a distinct cluster separate from genotypes reported thus far from Israel and Jordan and other Middle East variants. The *L. tropica* variants cluster together and are genetically homogeneous (on average, 122 single-nucleotide polymorphisms between samples), consistent with an outbreak-related scenario. We also found homozygous missense mutations or frameshifts in 14 genes reported to be involved in drug resistance, mostly antimony (Appendix Figure 3). That signature has not previously been reported in the *L. tropica* genome, which confirms the unique character of the samples from this region. Further work is required to understand the clinical effects of the discovery.

**Figure 2:**
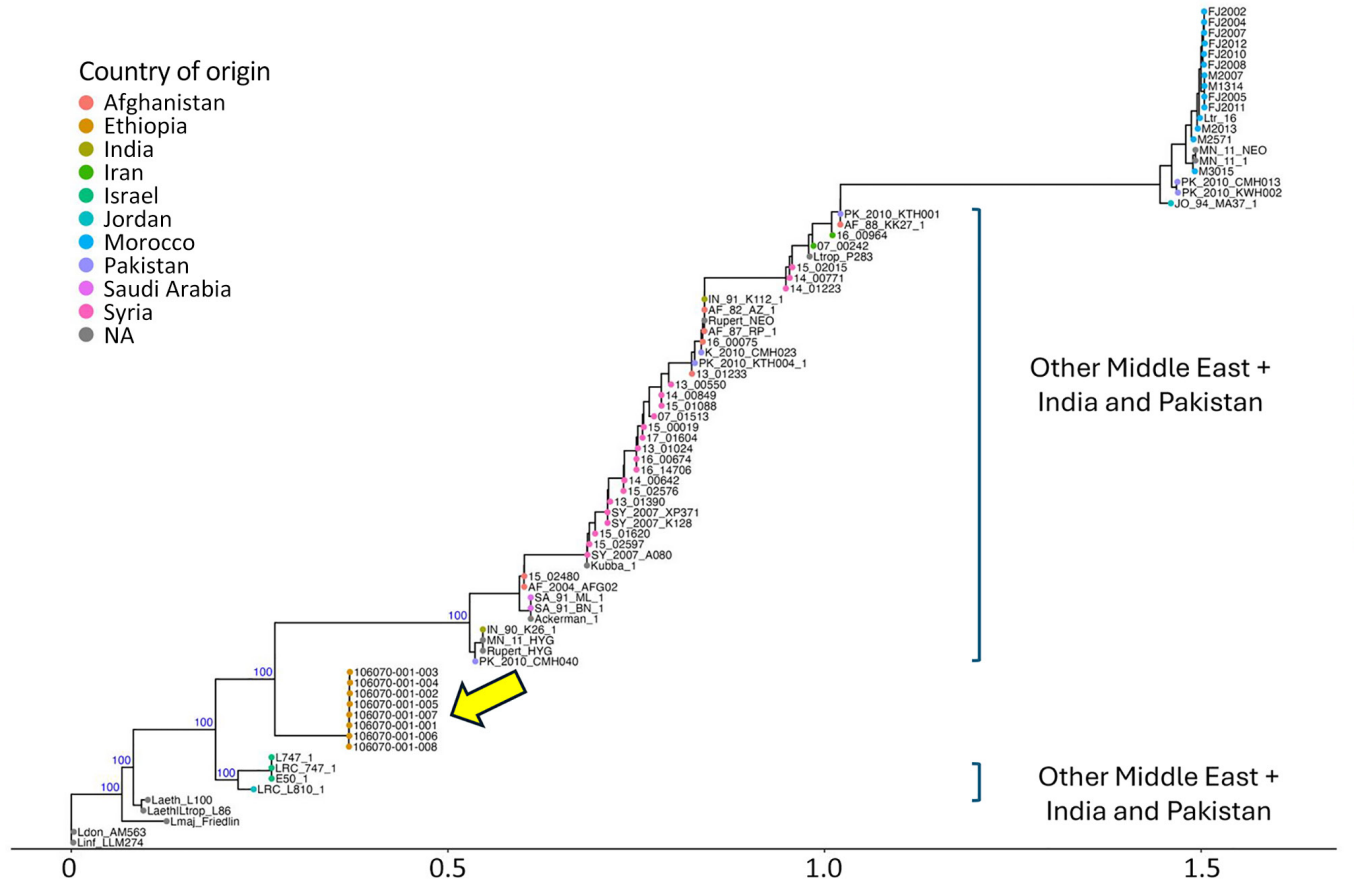
Rooted phylogenetic tree for genomic characterization of *Leishmania tropica* in cutaneous leishmaniasis, Somali region, Ethiopia, 2023. Phylogenetic tree of all publicly available *L. tropica* genomes (Appendix Table 3) was generated with RAxML (https://github.com/amkozlov/raxml-ng) with the general time reversible and gamma substitution model and showing close clustering of the 8 samples from Ethiopia (yellow arrow). Blue text indicates crucial bootstrap values. The *L. infantum* LLM274 genome was included as an outgroup. Scale bar indicates number of single-nucleotide polymorphism differences. NA, geographic origin of the *L. tropica* sample is unknown.

*L. tropica* is essentially endemic in Morocco, Turkey, Syria, Israel, Iraq, Azerbaijan, Iran, Uzbekistan, Afghanistan, Pakistan, and India (*8*). The broad distribution of *L. tropica* likely results from the anthroponotic nature of *L. tropica* transmission and the old communication axes in many of those countries, such as trade routes. In some regions, sporadic cases are reported, and the disease is thought to be zoonotic; possible animal reservoirs included hyraxes, bats, or wild rodents (*9*). The high genomic homogeneity in our sampled population shows the occurrence of an *L. tropica* outbreak in the Somali region of Ethiopia. We do not have the ability to trace whether the origin of the outbreak was a primary human case from which the parasite population spread or an animal reservoir. Nevertheless, this study highlights the risk for further expansion of the parasites from the human cases in the focus in the Somali region of Ethiopia. Public health officials should use genomic surveillance in humans, insect vectors, and animals in Ethiopia and neighboring countries, such as Kenya, where *L. tropica* was recently reported (*10*), to slow expansion of *L. tropica*.

Appendix Additional information for genomic characterization of *Leishmania tropica* in cutaneous leishmaniasis, Somali region, Ethiopia, 2023.
