# Supplementary material for: Genomic Characterization of Leishmania tropica in Cutaneous Leishmaniasis, Somali Region, Ethiopia, 2023
Source: Emerg Infect Dis. 2025 Jul;31(7):1483–6. doi: 10.3201/eid3107.241607 (PMC12205473; doi:10.3201/eid3107.241607)
Supplement: Appendix — Additional information for genomic characterization of Leishmania tropica in cutaneous leishmaniasis, Somali region, Ethiopia, 2023. [file 24-1607-Techapp-s1.pdf]

Article DOI: https://doi.org/10.3201/eid3107.241607

EID cannot ensure accessibility for supplementary materials supplied by authors. Readers who have difficulty accessing supplementary content should contact the authors for assistance.

Genomic Characterization of *Leishmania tropica* in Cutaneous Leishmaniasis, Somali Region, Ethiopia, 2023

Appendix

A1. Clinical Procedures and Data

This is a summary of clinical and epidemiologic data from patients (Appendix Table 1); more details can be found elsewhere (*1*).

Eight male members of the local militia who participated in the border conflict between the Somali and Afar regions were studied. The militias were local and deployed to the conflict area (border dispute) from both regions (Afar and Somali). Each referral facility had a dermatologist who examined suspected cases of cutaneous leishmaniasis (CL). The lesions were categorized as localized cutaneous leishmaniasis (LCL), or mucocutaneous leishmaniasis (MCL) (Appendix Table 1). Before starting treatment, fine needle aspirate (FNA) and skin scraping samples were taken from each case by experienced health professionals for Giemsa smear preparation. The Giemsa-stained results tested positive for leishmania amastigotes. Except for the two patients diagnosed at Duunyar Health Center, all six patients were treated with systemic intramuscular sodium stibogluconate (SSG) and were followed for 28 days. All six patients responded well to the SSG treatment, and no relapse cases have been reported from the treating facilities. The study was approved by the Institutional Review Boards of the EPHI (EPHI-IRB-554–2024), Institute of Tropical Medicine (ITM) Antwerp (ref 1745/24), and the University Hospital of Antwerp (ref 6229). Informed consent was obtained from all study participants. A material transfer agreement (MTA) was developed between EPHI and ITM to transfer DNA samples for species typing and genotyping"

A2. Laboratory Procedures

We received 37 samples, selected 8 (Appendix Table 2), using as criteria a minimum of 20 ng of input gDNA, and the % of Leishmania DNA at least 0.006% measured by qPCR as described elsewhere (*2*).

SureSelect (Agilent Technologies) was used to capture *Leishmania* genomic DNA following the standard SureSelect XTHS Target Enrichment System protocol for Illumina multiplexed sequencing platforms, using the *L. aethiopica* design (design ID S3373475). The probes for this design were developed in collaboration with Agilent Technologies, based on the *L. aethiopica* L147 reference genome (TriTrypDB version 54). Each probe is 120 nt in length, and the design follows a tiling strategy to ensure comprehensive coverage. In total, 249,945 probes were synthesized, targeting ≈30 Mbp of the genome. The probes are available from the authors upon request.

Our *L. aethiopica* array should be suitable for analyzing samples with both *L. aethiopica* and *L. tropica* infections. Indeed, *L. tropica* and *L. aethiopica* are phylogenetically highly related to each other (same species complex). Furthermore, we successfully applied an array developed using mostly *L. infantum* genome for capture and sequencing of *L. donovani* genome (also in a same species complex) (*2*).

The library preparation was done following SureSelect XT HS Target Enrichment System for Illumina Multiplexed Sequencing Platforms protocol (Agilent Technologies, Santa Clara, USA). First, samples with the starting concentration of the DNA between 0.7–3.9 ng/µl), were concentrated via purification on Ampure Beads and the elution in 10µl of low TE. In brief, quantity of 10–200 ng of input gDNA was fragmented using SureSelect Enzymatic Fragmentation kit (Agilent technologies, Santa Clara, USA). Subsequently, adaptor-ligated libraries were prepared using 8,10 or 12 number of cycles in pre-capture PCR, depending on the amount of input DNA. Libraries were hybridized with the custom probes at a dilution 1:10, and captured with Dynabeads MyOne Streptavidin T1 magnetic beads (Thermo Fisher Scientific, Waltham, USA). After washing steps, the DNA captured by streptavidin beads was amplified by PCR, and purified with AMPure XP beads. The quantity and quality of the libraries were assessed by TapeStation using High Sensitivity D1000 ScreenTape (Agilent Technologies, Santa

Clara, USA). Sequencing was done in Genomescan (Leiden, the Netherlands) using Illumina NovaSeq 6000 platform, 150 bp paired-end reads.

A3. Bioinformatic Procedures

Clinical samples for this study underwent PCR-free whole-genome sequencing using the Illumina NovaSeq platform, producing 2x150 bp paired-end reads. Additionally, we incorporated previously published sequencing datasets (Appendix Table 3), as available in the NCBI's Sequence Read Archive (SRA). This included *L. tropica* genomes from BioProjects PRJEB6281, PRJEB45563, and PRJNA978932, as well as genomes of *L. aethiopica* and other *Leishmania* species from BioProject PRJNA924694. The SRAtoolkit software was employed for downloading these publicly accessible sequencing data. Reads were aligned to the *L. tropica* L590 reference genome (TriTrypDB version 58) using BWA (v0.7.17) with a seed length of 50 (*3*). We selected only properly paired reads with a mapping quality score exceeding 30, processed using SAMtools (*4*). Duplicate reads were eliminated using the RemoveDuplicates feature in Picard software (v2.22.4). SNP calling followed the Genome Analysis ToolKit (GATK) best practices (v4.1.4.1). The procedure included: 1) Using GATK HaplotypeCaller to generate GVCF files for each sample. 2) Combining these GVCF files with the GATK CombineGVCF command. 3) Performing genotyping via GATK GenotypeGVCF. 4) Filtering SNPs and indels as per GATK's "best practices" using SelectVariants and VariantFiltration commands. For constructing phylogenetic trees, biallelic SNPs from VCF files were selected using BCFtools and converted to Phylip format with the vcf2phylip.py script. RAxML was employed with the GTR+G substitution model (https://github.com/amkozlov/raxml-ng), performing 100 bootstrap replicates. *L. infantum* JPCM5 was used as an outgroup. The resulting trees were visualized using ggtree for rooted phylogenetic trees and SplitsTree for unrooted phylogenetic networks.

A4. Competitive Mapping (for Species Identification)

Species identification was made by aligning -using BWA (*3*)- the sequencing reads from all eight samples to artificial concatenated genome consisting of 1) the human genome release GRCh38, 2) the *L. tropica* genome L590 as downloaded from TriTrypDB version 68, 3) the *L.*

aethiopica L147 reference genome as downloaded from TriTrypDB version 68. Using this competitive mapping approach, the BWA algorithm decides to which genome each sequencing reads maps with the highest quality. Using SAMtools (*4*), only those sequencing reads uniquely mapping to the artificial reference genome are selected. The relative percentage of reads mapping to either the *L. tropica* L590 or the *L. aethiopica* L147 reference genome is used to derive the species and exclude the potential hybrid nature of the strains. Based on this approach, all eight strains were identified as *L. tropica* (Appendix Figure 1).

A5. Mapping and Coverage Statistics

These are summarized in Appendix Table 4**.**

A6. Phylogenetics and Genomic Signatures of Drug Resistance

For the exploration of possible genomic signatures of drug resistance, we selected 11 loci that were previously shown to be involved in *L. tropica* resistance to antimonials, we complemented these with 4 loci reported to be involved in resistance to miltefosine and amphotericin B in other species. Relevant regions (indicated below) were subset using BCFtools (*10*). Only SNPs having an effect at the protein level (missense and non-sense mutations) were retained. Visualization of the heatmap (Appendix Figure 3) was performed using the pheatmap function in R. ***For antimonials***: Glutathione synthetase (GS) (*11*); Spermidine synthetase (SpS) (*11*); Thiol-dependant reductase (TDR) (*11*); Mitochondrial superoxide dismutase (SODA) (*12*); Glycosomal superoxide dismutase (SODB) (*12*); Tryparedoxin peroxidase (TryP) (*13*); Trypanothione reductase (TryR) (*13*); Aquaglyceroporine 1 (AQP1) (*14*); ABC transporter (MRPA) (*14*); Leishmania-activated C kinase gene (LACK1) (*15*); Amino acid permease (AAP3) (*16*). ***For miltefosine***; Miltefosine transporter (LdMT) (*17*); Beta-subunit of LdRos3 (*17*). ***For Amphotericine B***: Sterol C5-desaturase (C5D) (*18*); Sterol C24-methyltransferase (SMT) (*18*).

References

1. Abera A, Tadesse H, Beyene D, Geleta D, Abose E, Kinde S, et al. Outbreak of *Leishmania tropica* amongst militia members in a non-endemic district under conflict in the lowlands of the Somali Region in Ethiopia. https://www.medrxiv.org/content/10.1101/2024.10.05.24314933v1.

2. Domagalska MA, Imamura H, Sanders M, Van den Broeck F, Bhattarai NR, Vanaerschot M, et al. Genomes of *Leishmania* parasites directly sequenced from patients with visceral leishmaniasis in the Indian subcontinent. PLoS Negl Trop Dis. 2019;13:e0007900. PubMed https://doi.org/10.1371/journal.pntd.0007900

3. Li H, Durbin R. Fast and accurate short read alignment with Burrows-Wheeler transform. Bioinformatics. 2009;25:1754–60. PubMed https://doi.org/10.1093/bioinformatics/btp324

4. Li H, Handsaker B, Wysoker A, Fennell T, Ruan J, Homer N, et al.; 1000 Genome Project Data Processing Subgroup. The Sequence Alignment/Map format and SAMtools. Bioinformatics. 2009;25:2078–9. PubMed https://doi.org/10.1093/bioinformatics/btp352

5. Hadermann A, Heeren S, Maes I, Dujardin JC, Domagalska MA, Van den Broeck F. Genome diversity of *Leishmania aethiopica.* Front Cell Infect Microbiol. 2023;13:1147998. PubMed https://doi.org/10.3389/fcimb.2023.1147998

6. Talimi H, Daoui O, Bussotti G, Mhaidi I, Boland A, Deleuze JF, et al. A comparative genomics approach reveals a local genetic signature of *Leishmania tropica* in Morocco. Microb Genom. 2024;10:001230. PubMed https://doi.org/10.1099/mgen.0.001230

7. Iantorno SA, Durrant C, Khan A, Sanders MJ, Beverley SM, Warren WC, et al. Gene expression in *Leishmania* is regulated predominantly by gene dosage. MBio. 2017;8:e01393-17. PubMed https://doi.org/10.1128/mBio.01393-17

8. Glans H, Lind Karlberg M, Advani R, Bradley M, Alm E, Andersson B, et al. High genome plasticity and frequent genetic exchange in *Leishmania tropica* isolates from Afghanistan, Iran and Syria. PLoS Negl Trop Dis. 2021;15:e0010110. PubMed https://doi.org/10.1371/journal.pntd.0010110

9. Huson DH, Bryant D. Application of phylogenetic networks in evolutionary studies. Mol Biol Evol. 2006;23:254–67. PubMed https://doi.org/10.1093/molbev/msj030

10. Danecek P, Bonfield JK, Liddle J, Marshall J, Ohan V, Pollard MO, et al. Twelve years of SAMtools and BCFtools. Gigascience. 2021;10:giab008. PubMed https://doi.org/10.1093/gigascience/giab008

11. Valashani HT, Ahmadpour M, Naddaf SR, Mohebali M, Hajjaran H, Latifi A, et al. Insights into the trypanothione system in antimony-resistant and sensitive *Leishmania tropica* clinical isolates. Acta Trop. 2024;254:107190. PubMed https://doi.org/10.1016/j.actatropica.2024.107190

12. Bahrami A, Mohebali M, Reisi Nafchi H, Raoofian R, Kazemirad E, Hajjaran H. Overexpression of iron super oxide dismutases A/B genes are associated with antimony resistance of *Leishmania tropica* clinical isolates. Iran J Parasitol. 2022;17:473–82. PubMed https://doi.org/10.18502/ijpa.v17i4.11273

13. Nateghi-Rostami M, Tasbihi M, Darzi F. Involvement of tryparedoxin peroxidase (TryP) and trypanothione reductase (TryR) in antimony unresponsive of *Leishmania tropica* clinical isolates of Iran. Acta Trop. 2022;230:106392. PubMed https://doi.org/10.1016/j.actatropica.2022.106392

14. Mohebali M, Kazemirad E, Hajjaran H, Kazemirad E, Oshaghi MA, Raoofian R, et al. Gene expression analysis of antimony resistance in *Leishmania tropica* using quantitative real-time PCR focused on genes involved in trypanothione metabolism and drug transport. Arch Dermatol Res. 2019;311:9–17. PubMed https://doi.org/10.1007/s00403-018-1872-2

15. Hajjaran H, Kazemi-Rad E, Mohebali M, Oshaghi MA, Khadem-Erfan MB, Hajaliloo E, et al. Expression analysis of activated protein kinase C gene (LACK1) in antimony sensitive and resistant *Leishmania tropica* clinical isolates using real-time RT-PCR. Int J Dermatol. 2016;55:1020–6. PubMed https://doi.org/10.1111/ijd.13321

16. Kazemi-Rad E, Mohebali M, Khadem-Erfan MB, Hajjaran H, Hadighi R, Khamesipour A, et al. Overexpression of ubiquitin and amino acid permease genes in association with antimony resistance in *Leishmania tropica* field isolates. Korean J Parasitol. 2013;51:413–9. PubMed https://doi.org/10.3347/kjp.2013.51.4.413

17. Pérez-Victoria FJ, Sánchez-Cañete MP, Castanys S, Gamarro F. Phospholipid translocation and miltefosine potency require both *L. donovani* miltefosine transporter and the new protein LdRos3 in *Leishmania* parasites. J Biol Chem. 2006;281:23766–75. PubMed https://doi.org/10.1074/jbc.M605214200

18. Pountain AW, Weidt SK, Regnault C, Bates PA, Donachie AM, Dickens NJ, et al. Genomic instability at the locus of sterol C24-methyltransferase promotes amphotericin B resistance in *Leishmania* parasites. PLoS Negl Trop Dis. 2019;13:e0007052. PubMed https://doi.org/10.1371/journal.pntd.0007052

Appendix Table 1. Clinical data of the 8 patients studied*

Specimen ID	Sex	Age	Facility name	Site of lesion	Number of lesions	Types of CL	Duration since onset of lesion in months	Giemsa stained result
WH08	Male	40	JUSYCSH	Face	12	LCL	6	Positive
WH09a	Male	20	JUSYCSH	Face, Nose, Lips	13	MCL	8	Positive
WH11	Male	22	JUSYCSH	Face	8	LCL	6	Positive
WH12	Male	40	JUSYCSH	Face, Nose, Lips	14	MCL	7	Positive
S04	Male	29	DHC	legs	3	LCL	6	Positive
S06	Male	42	DHC	Face, Nose, Lips	18	MCL	6	Positive
S12	Male	36	SPH	Face, Nose, Lips	11	MCL	5	Positive
WH15	Male	21	JUSYCSH	Face	10	LCL	6	Positive

*DHC, Duunyar Health Center; JUSYCSH, Jigjiga University Sheik Hassen Yabare Comprehensive Specialized Hospital; SPH, Sitti Primary Hospital.

Appendix Table 2. Technical features of the samples studied

Samples	Total quantity of DNA (ng)	% Leishmania DNA	Enrichment
WH08	6860	0,016	no
WH09a	1981	0,024	no
WH11	1071	0,053	no
WH12	1089	0,014	no
S04	51	0,152	Ampure Beads
S06	117	0,438	Ampure Beads
S12	22	0,081	Ampure Beads
WH15	165	0,014	Ampure Beads

Appendix Table 3. List of previously published genome sequences used*

Species	Reference	Workname	Country
L. aethiopica	(*5*)	Laeth_L100	
L. aethiopica/L. tropica	(*5*)	Laeth|Ltrop_L86	
L. donovani	(*5*)	Ldon_AM563	
L. infantum	(*5*)	Linf_LLM274	
L. major	(*5*)	Lmaj_Friedlin	
L. tropica	(*5*)	Ltrop_P283	Israel
New sample	This study	106070–001–001	Ethiopia
New sample	This study	106070–001–002	Ethiopia
New sample	This study	106070–001–003	Ethiopia
New sample	This study	106070–001–004	Ethiopia
New sample	This study	106070–001–005	Ethiopia
New sample	This study	106070–001–006	Ethiopia
New sample	This study	106070–001–007	Ethiopia
New sample	This study	106070–001–008	Ethiopia
L. tropica	PRJNA978932 (*6*)	FJ2002	Morocco
L. tropica	PRJNA978932 (*6*)	FJ2004	Morocco
L. tropica	PRJNA978932 (*6*)	FJ2005	Morocco
L. tropica	PRJNA978932 (*6*)	FJ2007	Morocco
L. tropica	PRJNA978932 (*6*)	FJ2008	Morocco
L. tropica	PRJNA978932 (*6*)	FJ2010	Morocco
L. tropica	PRJNA978932 (*6*)	FJ2011	Morocco
L. tropica	PRJNA978932 (*6*)	FJ2012	Morocco
L. tropica	PRJNA978932 (*6*)	Ltr_16	Morocco
L. tropica	PRJNA978932 (*6*)	M1314	Morocco
L. tropica	PRJNA978932 (*6*)	M2007	Morocco
L. tropica	PRJNA978932 (*6*)	M2013	Morocco
L. tropica	PRJNA978932 (*6*)	M2571	Morocco
L. tropica	PRJNA978932 (*6*)	M3015	Morocco
L. tropica	PRJEB6281 (*7*)	Ackerman_1	na
L. tropica	PRJEB6281 (*7*)	E50_1	Israel
L. tropica	PRJEB6281 (*7*)	Kubba_1	na
L. tropica	PRJEB6281 (*7*)	L747_1	Israel
L. tropica	PRJEB6281 (*7*)	AF_2004_AFG02	Afghanistan
L. tropica	PRJEB6281 (*7*)	AF_82_AZ_1	Afghanistan
L. tropica	PRJEB6281 (*7*)	AF_87_RP_1	Afghanistan
L. tropica	PRJEB6281 (*7*)	AF_88_KK27_1	Afghanistan
L. tropica	PRJEB6281 (*7*)	LRC_L810_1	Jordan

Species	Reference	Workname	Country
L. tropica	PRJEB6281 (*7*)	LRC_747_1	Israel
L. tropica	PRJEB6281 (*7*)	IN_90_K26_1	India
L. tropica	PRJEB6281 (*7*)	IN_91_K112_1	India
L. tropica	PRJEB6281 (*7*)	JO_94_MA37_1	Jordan
L. tropica	PRJEB6281 (*7*)	PK_2010_CMH013	Pakistan
L. tropica	PRJEB6281 (*7*)	K_2010_CMH023	Pakistan
L. tropica	PRJEB6281 (*7*)	PK_2010_CMH040	Pakistan
L. tropica	PRJEB6281 (*7*)	PK_2010_KTH001	Pakistan
L. tropica	PRJEB6281 (*7*)	PK_2010_KTH004_1	Pakistan
L. tropica	PRJEB6281 (*7*)	PK_2010_KWH002	Pakistan
L. tropica	PRJEB6281 (*7*)	SA_91_BN_1	Saudi Arabia
L. tropica	PRJEB6281 (*7*)	SA_91_ML_1	Saudi Arabia
L. tropica	PRJEB6281 (*7*)	SY_2007_A080	Syria
L. tropica	PRJEB6281 (*7*)	SY_2007_K128	Syria
L. tropica	PRJEB6281 (*7*)	SY_2007_XP371	Syria
L. tropica	PRJEB6281 (*7*)	MN_11_HYG	na
L. tropica	PRJEB6281 (*7*)	MN_11_NEO	na
L. tropica	PRJEB6281 (*7*)	MN_11_1	na
L. tropica	PRJEB6281 (*7*)	Rupert_HYG	na
L. tropica	PRJEB6281 (*7*)	Rupert_NEO	na
L. tropica	PRJEB45563 (*8*)	07_00242	Iran
L. tropica	PRJEB45563 (*8*)	07_01513	Syria
L. tropica	PRJEB45563 (*8*)	13_00550	Syria
L. tropica	PRJEB45563 (*8*)	13_01024	Syria
L. tropica	PRJEB45563 (*8*)	13_01233	Afghanistan
L. tropica	PRJEB45563 (*8*)	13_01390	Syria
L. tropica	PRJEB45563 (*8*)	14_00642	Syria
L. tropica	PRJEB45563 (*8*)	14_00771	Syria
L. tropica	PRJEB45563 (*8*)	14_00849	Syria
L. tropica	PRJEB45563 (*8*)	14_01223	Syria
L. tropica	PRJEB45563 (*8*)	15_00019	Syria
L. tropica	PRJEB45563 (*8*)	15_01088	Syria
L. tropica	PRJEB45563 (*8*)	15_01620	Syria
L. tropica	PRJEB45563 (*8*)	15_02015	Syria
L. tropica	PRJEB45563 (*8*)	15_02480	Afghanistan
L. tropica	PRJEB45563 (*8*)	15_02576	Syria
L. tropica	PRJEB45563 (*8*)	15_02597	Syria
L. tropica	PRJEB45563 (*8*)	16_00075	Afghanistan
L. tropica	PRJEB45563 (*8*)	16_00674	Syria
L. tropica	PRJEB45563 (*8*)	16_00964	Iran
L. tropica	PRJEB45563 (*8*)	16_14706	Syria
L. tropica	PRJEB45563 (*8*)	17_01604	Syria

*na, not available; PRJxxx, BioProjects.

Appendix Table 4. Summary of mapping and coverage statistics for the eight novel samples analyzed in this study*

			Filtering steps			% of genome covered			Coverage stats	
Sample	Raw reads	Mapped reads	MapQ >30	Duplicate removal	Proper paired	cov >5x	cov >10x	cov >25x	Median coverage	Mean coverage
106070–001–001	27,701,534	11,366,087	10,777,137	3,576,927	3,531,687	76.3%	69.0%	47.2%	23	28.1
106070–001–002	26,594,318	11,461,438	10,873,377	3,700,731	3,645,914	76.7%	69.7%	48.6%	24	28.7
106070–001–003	23,651,482	8,514,150	8,101,035	4,483,757	4,426,731	79.6%	74.0%	57.7%	32	36.7
106070–001–004	22,727,278	6,323,439	6,011,241	1,367,767	1,345,891	66.4%	47.6%	8.4%	9	11.1
106070–001–005	41,830,239	27,387,137	25,963,829	12,397,256	12,112,044	80.8%	78.1%	71.6%	80	91.3
106070–001–006	52,131,039	43,262,584	41,127,372	25,412,697	25,010,886	83.8%	81.6%	77.7%	172	193.6
106070–001–007	42,903,563	23,986,132	22,827,208	5,767,168	5,669,680	82.8%	79.0%	68.9%	46	45.6
106070–001–008	63,238,068	13,167,852	12,449,758	2,177,001	2,143,931	74.2%	63.6%	26.0%	15	17.1

*The first five columns show the number of paired reads retained after consecutive filtering steps (*1*): input data (*2*), reads mapped to the *L. tropica* reference genome (*3*), reads with a BWA mapping quality score higher than 30 (*4*), reads remaining after duplicate removal, and (*5*) properly paired reads. The next three columns indicate the percentage of the genome covered at least 5x, 10x, and 25x, respectively. The final two columns report the median and mean coverage per sample.

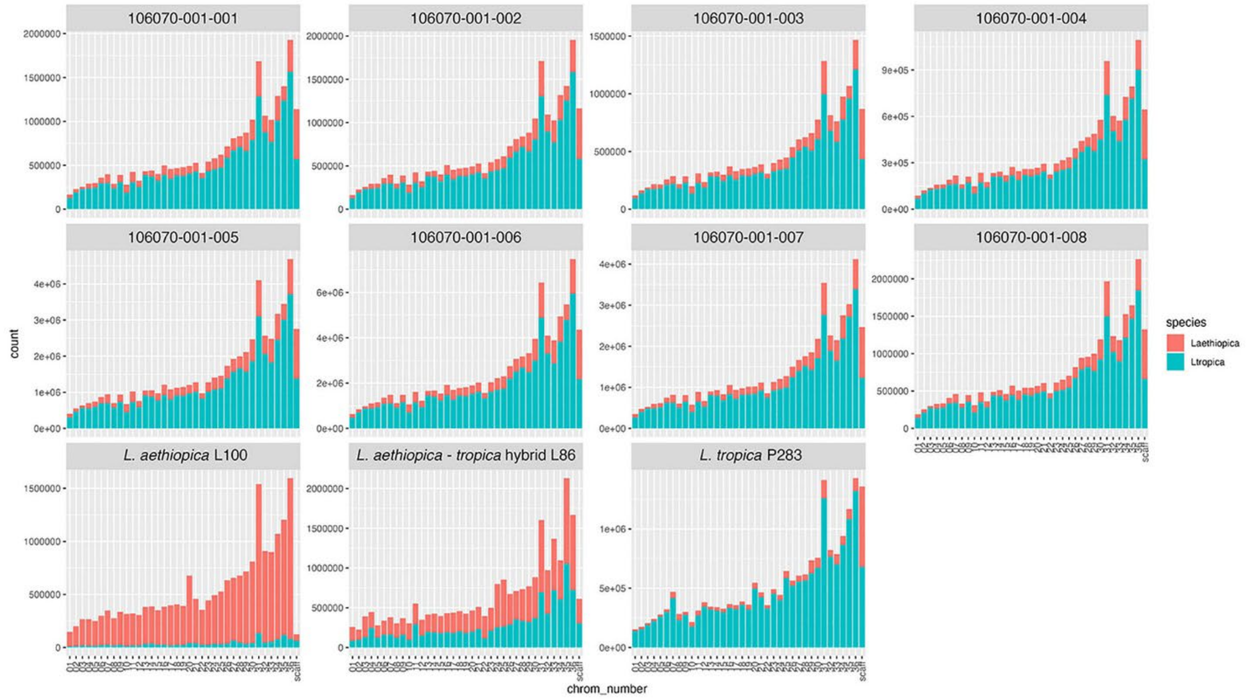

Appendix Figure 1. Competitive mapping results using a combined reference genome of *L. tropica* L590, *L. aethiopica* L147, and the human genome GRCh38. The first two rows represent the eight sequencing datasets generated in this study. The third row includes benchmark data from a *L. aethiopica* strain (L100), a *L. aethiopica/L. tropica* hybrid (L86), and a *L. tropica* strain (P283). For each strain, the number of reads mapped to each chromosome is shown, with red bars representing reads mapping to *L. aethiopica* and green bars representing reads mapping to *L. tropica*.

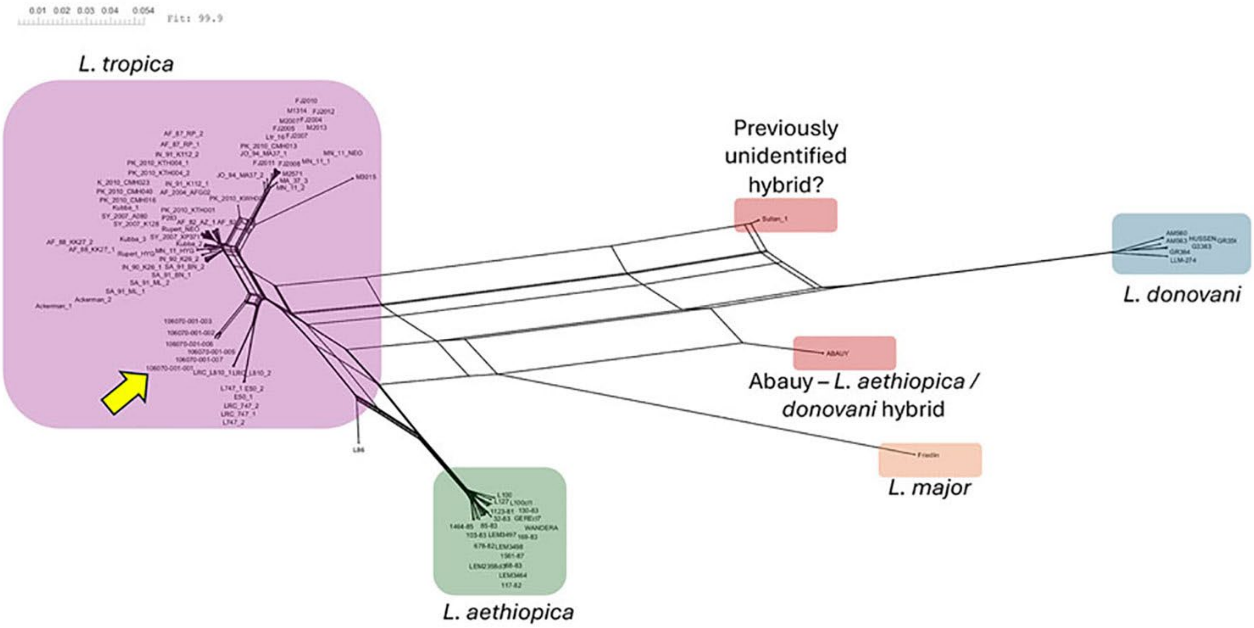

Appendix Figure 2. Phylogenetic network indicating the phylogenetic relation between a subset of different Leishmania genomes (strains belonging to *L. tropica*, *L. aethiopica*, *L. major*, and *L. donovan*i). The quality-filtered SNP VCF files were transformed into FASTA format using the vcf2fasta.py script (accessible at github.com/FreBio/mytools/blob/master/vcf2fasta.py). To explore the phylogenetic relationships among the genomes, a phylogenetic network was generated with SplitsTree version 4.19.0 (*9*) based on concatenated bi-allelic SNPs. The eight newly sequenced genomes (yellow arrow) group together with the other *L. tropica* genomes. Apart from a previously identified hybrid between *L. aethiopica* and *L. donovani* (Abauy strain), we also found a potential previously unidentified hybrid based on its position in this phylogenetic network (Sultan_1)

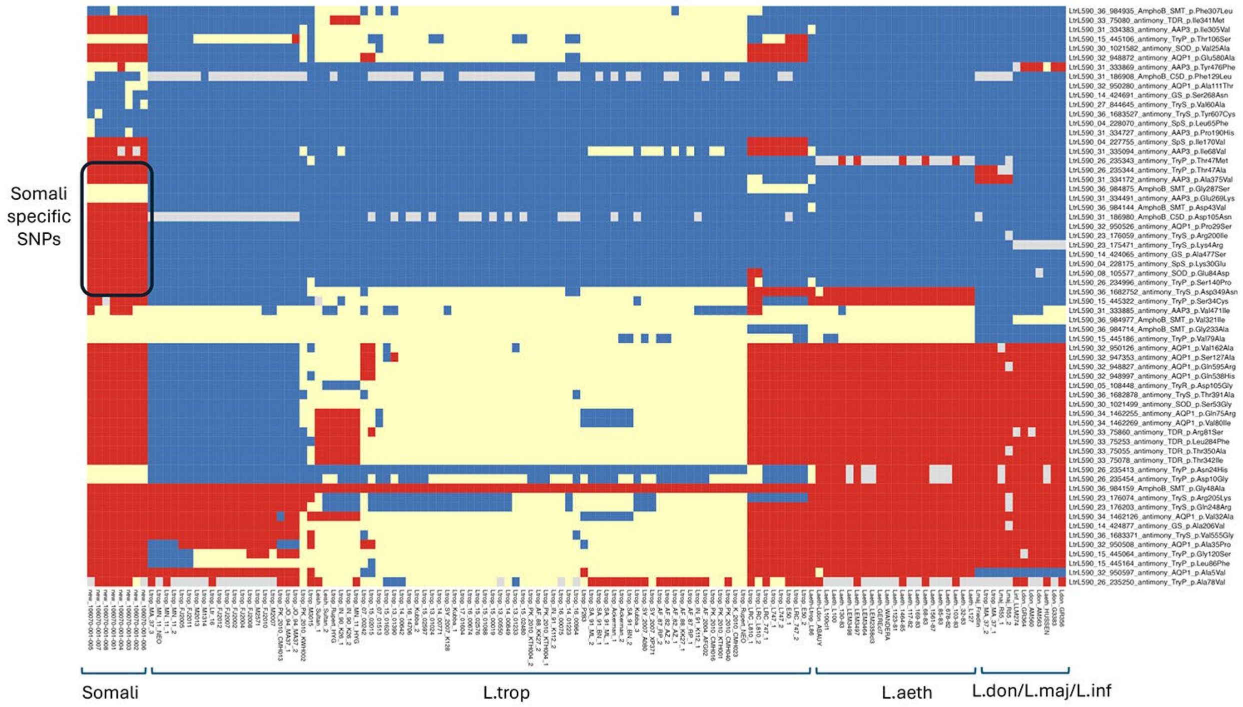

Appendix Figure 3. Heatmap for genomic characterization of *Leishmania tropica* in cutaneous leishmaniasis, Somali Region, Ethiopia, 2023. Distribution of SNPs in genes reported to be associated with drug-resistant phenotypes. Color scheme represents different SNP categories: blue, absence of SNPs; yellow, heterozygous SNPs; red, homozygous SNPs. Naming convention for SNPs: gene of interest, position in genome, type of mutation, and its effect on corresponding protein. L.aeth, *L. aethiopica*; L.don, *L. donovani*; L.inf, *L. infantum*; L.maj, *L. major*; L.trop, *L. tropica*; SNP, single-nucleotide polymorphism.
